# Intravitreal and Subconjunctival Melphalan for Retinoblastoma in Transgenic Mice

**DOI:** 10.1155/2014/829879

**Published:** 2014-03-10

**Authors:** Nisha V. Shah, D. G. Pham, T. G. Murray, C. Decatur, E. Hernandez, Nikesh N. Shah, M. Cavalcante, S. K. Houston

**Affiliations:** ^1^Department of Ophthalmology, Tulane University, New Orleans, LA, USA; ^2^Bascom Palmer Eye Institute, University of Miami Miller School of Medicine, P.O. Box 016880, Miami, FL 33101, USA; ^3^Murray Ocular Oncology and Retina, Miami, FL, USA; ^4^Wills Eye Hospital, Retina Service, Philadelphia, PA, USA

## Abstract

*Purpose*. To measure the chemotherapeutic effects of focal melphalan (intravitreal and subconjunctival) on tumor burden, hypoxia, and vasculature in LHBETATAG murine retinoblastoma model. *Methods.* LHBETATAG transgenic mice were treated with a single 1 mcg intravitreal injection of melphalan, 100 mcg subconjunctival injection, or semiweekly 10 mcg subconjunctival injections for 3 weeks. At 1 or 3 weeks, eyes were enucleated, serially sectioned, and processed with haematoxylin and eosin (H&E) for tumor burden measurements and probed with immunofluorescence to analyze tumor hypoxia and vasculature. *Results*. Focal melphalan significantly reduced retinal tumor size (*P* < 0.02) when given intravitreally or subconjunctivally. Eyes treated with a one-time intravitreal injection of 1 mcg melphalan had significantly smaller tumors at both 1 week (*P* = 0.017) and at 3 weeks after injection (*P* = 0.005). Intratumoral hypoxia showed a significant decline in hypoxia at 1 week following intravitreal injection and after maximum dosage of subconjunctival melphalan. Total vasculature was not significantly affected following intravitreal administration. *Conclusion*. Focal delivery of melphalan via intravitreal or subconjunctival injection has a significant effect on reducing tumor burden, hypoxia, and vasculature, in the treatment of murine retinoblastoma tumors.

## 1. Introduction

Over the past decade, systemic chemotherapy combined with focal consolidative treatment has gained popularity as the initial treatment of choice for retinoblastoma, due to the benefits of globe preservation and the potential to maintain some functional vision—an important factor given the long remaining lifespan of retinoblastoma survivors [1, 2]. Previously, external beam radiation therapy (EBRT) served as the primary globe-salvaging treatment, but it is now rarely administered due to radiation-induced side effects [3–5].

Local treatment forms include subconjunctival (sub-Tenons') injections [6], intravitreal injections [7–10], and intra-arterial administration [11–13]. Melphalan is already being used in the clinical setting in the form of intra-arterial delivery and intravitreal injections for treatment of retinoblastoma, and preliminary studies have shown promise [14]. One of the largest studies on intra-arterial chemotherapy included 95 eyes with retinoblastoma. The authors report globe salvage of 70% for all eyes at 2 years, with eyes treated with intra-arterial chemotherapy as primary management having a higher success rate (81.7%) compared to salvage (58.4%) [11]. A study at Bascom Palmer on 12 eyes of 10 children with advanced RB (Reese-Ellsworth stage Vb or International Classification Group D) [12] demonstrated that eyes receiving ophthalmic artery melphalan for 9 months showed no tumor progression at the 6-month follow-up visit. Furthermore, in severe cases requiring enucleation, infusing melphalan directly into the ophthalmic artery significantly reduced the enucleation rate from 100% to 23.5% [13]. A recent study by Munier et al. [14] reported on the efficacy of intravitreal melphalan for advanced retinoblastoma with vitreous seeding. Twenty-three patients received a total of 122 intravitreal injections of melphalan (dose: 20–30 mcg) using a specialized technique and strict patient selection criteria. The authors showed that globe salvage was 87%. Ghassemi and Shields reported on 12 cases treated with intravitreal melphalan for vitreous seeding in retinoblastoma. Patients received doses of 8–50 mcg of melphalan, with tumor control of 60% at 6 months [15]. These studies suggest that melphalan could be utilized as a globe-conserving treatment option in advanced RB cases [11, 13, 15].

The advantages of administering chemotherapeutic agents locally, specifically subconjunctival (sub-Tenons') [6] or intravitreal injections [6], include the ability to administer high concentrations of medication that would otherwise cause considerable toxicity if administered systemically. The delivery of melphalan through the intra-arterial route is a clinically practiced therapy for treatment of retinoblastoma [11, 12, 16–18], but it has been shown to cause multiple adverse effects locally. These include microemboli formation, choroidal infarction, salt-and-pepper retinopathy, vitreous hemorrhage, myositis, lid edema, forehead hyperemia, eyelash loss as well as neutropenia, and peripapillary cotton wool spots [12, 19–23]. Other vascular side effects include ophthalmic artery stenosis and potentially blinding vascular obstruction from thrombotic events [21–23]. A report by Shields et al. has shown potential permanent and blinding complications of ophthalmic artery stenosis, retinal artery occlusion [22], and other reports of ciliary thrombosis in enucleated eyes receiving IAC [22, 23].

These findings suggest the need for further research to determine ideal dosing and delivery strategies [23]. We have previously reported that local chemotherapeutic carboplatin through intravitreal or subconjunctival/sub-Tenon's injections in murine models resulted in dose-dependent effects on tumor control [6, 7]. One study showed complete tumor regression in 2/6 mice 15 days following subretinal injection of melphalan (500 *μ*g/kg) [24]. Although intraocular retinoblastoma is increasingly treated with focally delivered melphalan and other chemotherapeutic agents, little research has been done showing the efficacy of these local treatments (intravitreal and subconjunctival melphalan) on tumor burden, vasculature, and hypoxia, in animal models.

This study utilizes the transgenic *LH*⁡_BETA_T_AG_ mouse with hereditary retinoblastoma, which has been characterized thoroughly and appears similar to human retinoblastoma in anatomic, genetic, light, and electron microscopic, immunohistochemical, and ultrastructural features [25–28]. This study's aims are to explore the efficacy of intravitreal and subconjunctival melphalan in controlling intraocular retinoblastoma and its mechanism of action.

## 2. Methods

All experiments in this study were conducted in accordance with the ARVO Statement for the Use of Animals in Ophthalmic and Vision Research and the University of Miami institutional guidelines regarding animal experimentation. The study protocol was approved by the University of Miami Institutional Animal Care and Use Review Board Committee.

### 2.1. Subconjunctival and Intravitreal Injections of Melphalan

The *LH*⁡_BETA_T_AG_ transgenic mouse model used in this study has been characterized previously [26, 27, 29, 30]. This animal model develops bilateral multifocal retinal tumors that are stable and grow at a predictable rate (i.e., tumor at 4 weeks is undetectable, at 8 weeks is small, at 12 weeks is medium, and at 16 weeks is large) [31]. Offspring bearing the transgene were identified by polymerase chain reaction analysis of tail DNA. Animals were anesthetized with intraperitoneal injections of ketamine and xylazine before intravitreal and/or subconjunctival treatment injections.

Melphalan for injection was prepared by rapidly injecting 10 mL of the diluent (sodium citrate 0.2 g, propylene glycol 6.0 mL, ethanol (96%) 0.52 mL, and water) [32] directly into 50 mg of melphalan and vigorously shaken. One mL of this 5 mg/mL solution was diluted to 0.5 *μ*g/mL using 0.9% normal saline solution to prepare a 10 *μ*g/20 *μ*L solution. From the 5 mg/mL solution, we also derived 20 *μ*L to achieve a 100 *μ*g/20 *μ*L solution. Melphalan was administered within 1 hour of preparation for all cases.

Twelve-week-old *LH*⁡_BETA_T_AG_ retinoblastoma tumor eyes (*n* = 18) were treated with either 10 *μ*g per 20 *μ*L subconjunctival melphalan 2 times a week for 3 weeks (*n* = 8) or with a single administration of 1 *μ*g per 2 *μ*L intravitreal melphalan (*n* = 10). The intravitreal injections were administered using a 33-gauge needle inserted 1 mm posterior to the limbus and directed posterior to the lens under direct visualization through the pupil. Subconjunctival injections were delivered with a 33-gauge needle inserted into the nasal and superior subconjunctival space. A microvolume delivery pump was used to ensure accurate and reproducible delivery of the 2 *μ*L or 20 *μ*L volumes. Litter matched animals that served as controls were administered either 2 *μ*L or 20 *μ*L of vehicle control for the intravitreal and subconjunctival treatment groups, respectively. Mice treated with intravitreal melphalan were sacrificed after 1 week (*n* = 6) or 3 weeks of therapy (*n* = 4). A third treatment arm included 17-week-old *LH*⁡_BETA_T_AG_ retinoblastoma tumor eyes (*n* = 7) that were given one administration of 100 *μ*g per 20 *μ*L subconjunctival melphalan and sacrificed after 3 weeks.

Mice were euthanized with CO_2_ fumes and eyes were enucleated. Tumor sections were analyzed for tumor burden, hypoxia, and vasculature.

### 2.2. Histopathologic Study of Transgenic Mouse Tumors

Eyes were sectioned serially and processed for standard hematoxylin and eosin (H&E) staining. Microscopic images of H&E-stained sections (8 *μ*m) were obtained with a digital camera at 40x magnification. Tissue sections containing the largest cross-sectional tumor area were chosen for analysis. Tumor boundaries were manually traced using imaging software (Image Pro Express Software; Media Cybernetics, Silver Spring, MD). Tumor areas for all eyes were averaged, yielding a mean area for each treatment group. Tumor burden was expressed as the pixel area of neoplastic tissue using Adobe Photoshop Premiere Pro (2006).

### 2.3. Measuring Hypoxic Regions

To assess tumor hypoxia after treatment, *LH*⁡_BETA_T_AG_ mice received intraperitoneal injections of 200 *μ*L of pimonidazole (10 mg/mL; Chemicon, Temecula, CA). Pimonidazole binds thiol-containing proteins in cells under low O_2_ tension [31]. These adducts can be detected with specific antibodies and stained using immunohistochemical techniques. Animals were euthanized 2 hours after pimonidazole injection, and eyes were harvested and sectioned for histopathologic examination. Eye sections were fixed with cold methanol for 10 minutes, then immunostained with a hypoxia-specific FITC-labeled antibody recognizing pimonidazole adducts (Hypoxyprobe 1-Mab-1-FITC, clone 4.3.11.3; Chemicon) and cell nuclei-specific 4′, 6′ diamidino-2-phenylindole (DAPI, 1 : 5,000; Invitrogen, Carlsbad, CA). The density of hypoxia was measured by calculating the ratio of the amount of pixels stained with pimonidazole (i.e., marker for hypoxia) over the amount of pixels for total tumor area (100 × HPF; Adobe Photoshop Premiere Pro; Adobe, San Jose, CA).

### 2.4. Measuring Tumor Vasculature

Tumor samples were frozen in Optimal Cutting Temperature solution (Tissue-Tek, Sakura Finetek, Torrance, CA) shortly after enucleation and were serially sectioned (8 *μ*m). Slides were fixed with chilled methanol for 10 min at −20°C before immunohistochemical staining. Total vessels were detected with biotin conjugated lectin (1 : 500 L3759; Sigma Chemical Co, St. Louis, MO) which specifically binds to pericytes [33], followed by extrAvidin-cy3 for labeling (1 : 500; E4142' Sigma Chemical Co, St. Louis, MO). Neovessels were probed using anti-endoglin monoclonal antibody (anti-CD105, 1 : 500; sc18893; Santa Cruz Biotechnology, Inc., Santa Cruz, CA), which has specificity for vascular endothelial cells undergoing angiogenesis [34]. Alexa Fluor 488-conjugated secondary antibody was then used (anti-IGg; 1 : 500; A21208; Invitrogen, Carlsbad, CA) to detect the anti-endoglin antibody. Omission of the primary antibody (secondary only) was used as a negative control for nonspecific binding. Cell nuclei were stained for 5 minutes with 4′, 6′ diamidino-2-phenylindole (DAPI, 1 : 5000; Invitrogen). Blood vessel caliber analysis and grading were performed as previously described [35]. Cross-sections of eyes containing tumors were examined for the presence of the described markers with a BX51 Olympus upright fluorescence microscope (Olympus American Inc., Melville, NY). All fluorescent images were obtained at 200x magnification and quantified by measuring cross sectional area.

### 2.5. Light and Fluorescent Microscopy

Cross-sections of eyes containing tumors were examined for the presence of the described markers with a BX51 Olympus upright fluorescence microscope (Olympus American Inc., Melville, NY). All fluorescent images were obtained at 200x magnification using controlled filters to visualize DAPI, (mamely. Alexa Fluor 488, and 568 signals). Light micrographs of tumor burden were imaged at 40X with the Olympus SZH10 stereo microscope.

### 2.6. Statistical Methods

Analysis of variance followed by two-sample *t*-test was used to evaluate differences between treatment groups with respect to tumor burden, hypoxia, and vasculature. Results were reported from untransformed data with square root *P* values. The mean reductions of vessels, hypoxia, and tumor burden after melphalan treatment from the vehicle control were evaluated by one- and two-sample *t*-test. Values were considered significant with *P* ≤ 0.05.

## 3. Results

**Figure 1:**
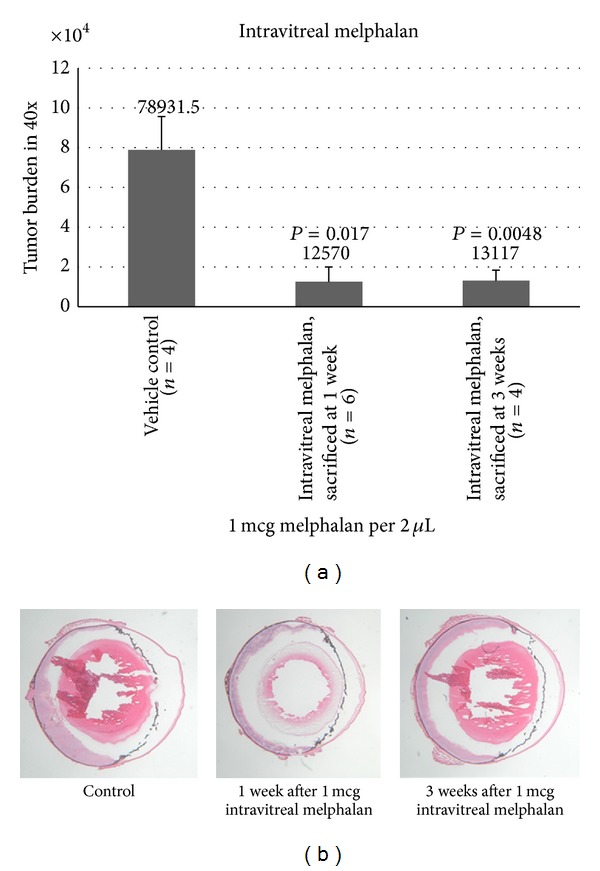
Mean tumor burden (40x) for eyes at 1 week and 3 weeks following intravitreal injection of 1 mcg melphalan. Tumor inhibition was statistically significant after 1 week and after 3 weeks following intravitreal treatment.

Histopathologic examination at 1 week and 3 weeks after treatment revealed that intravitreal melphalan 1 *μ*g/2 uL significantly reduced tumor burden compared to untreated control eyes (Figure 1). Furthermore, three treated eyes had complete absence of tumor on histopathologic examination at 1 week (*n* = 2) and 3 weeks (*n* = 1) following intravitreal melphalan treatment. One week after intravitreal melphalan therapy, tumor burden was significantly reduced by 85% compared to control (*P* = 0.017); three weeks after treatment with a single injection, tumor burden remained significantly reduced by 83% (*P* = 0.0048).

**Figure 2:**
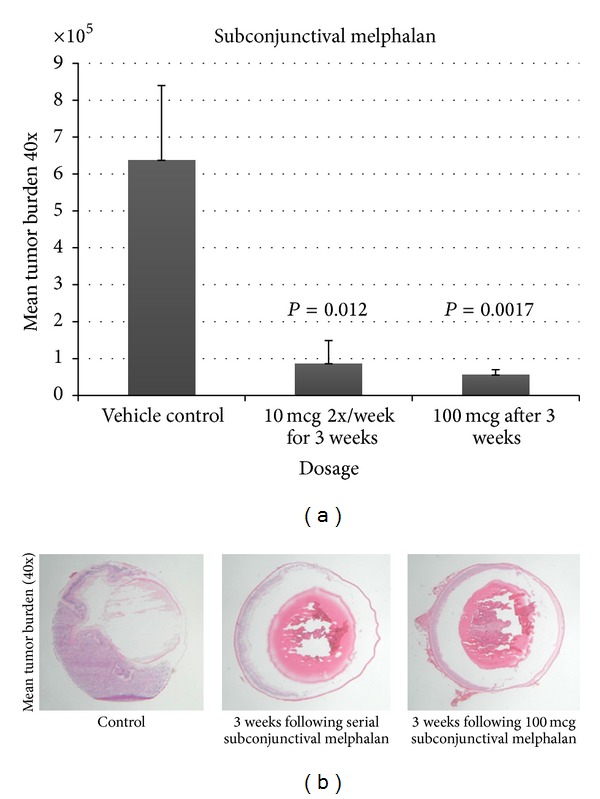
Mean tumor burden (40x) following serial subconjunctival injections of 10 mcg/2x a week for 3 weeks and following 1-time administration of 100 *μ*g of melphalan. Both treatment groups demonstrated statistically significant decline in tumor burden.

Subconjunctival injection of melphalan also significantly reduced tumor burden in transgenic retinoblastoma mice (Figure 2). Ten *μ*g of melphalan administered 2 times a week over a 3-week period (total of 6 treatments) showed a decrease in tumor burden of 86% compared to controls (*P* = 0.012), while a one-time administration of 100 *μ*g of melphalan in advanced tumors (17 weeks old) significantly decreased tumor burden by 91% compared to controls (*P* = 0.0017). No toxicities were microscopically seen following treatments.

**Figure 3:**
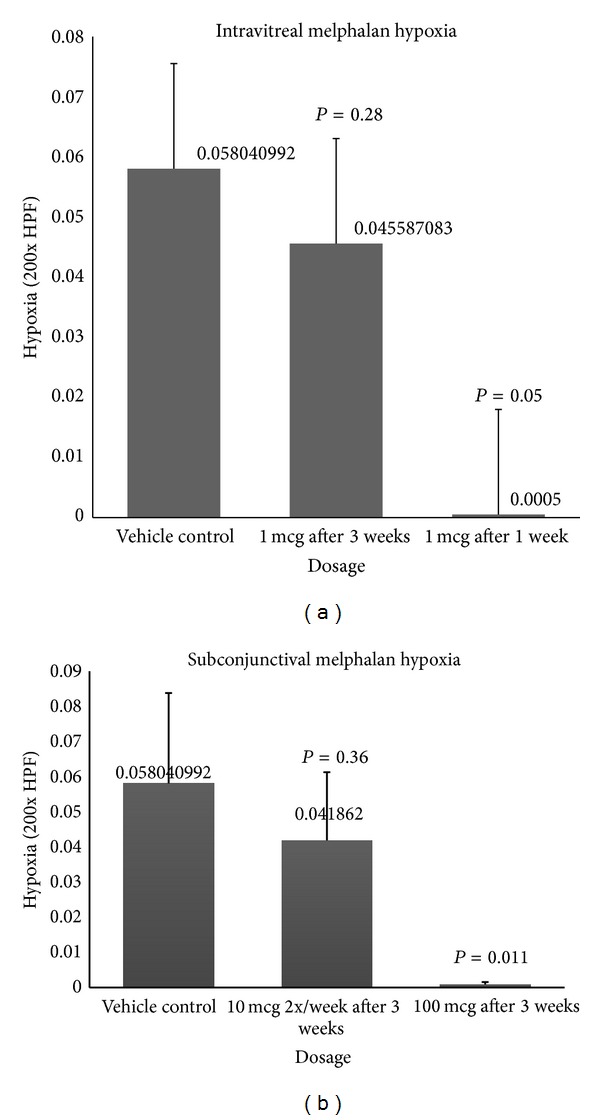
Hypoxia was significantly reduced 1 week after therapy with 1 *μ*g intravitreal melphalan and after administration of maximum concentration of subconjunctival melphalan.

Hypoxia (measured in percentage of hypoxia/area of tumor) was seen at a level of 5.8%/square area in control eyes and was significantly reduced by >99% one week following intravitreal melphalan treatment (*P* = 0.05). A significant decline in hypoxia was not observed after three weeks; however, hypoxia remained reduced, but only at 21.5% (*P* = 0.28) (Figure 3). A single 100 *μ*g subconjunctival injection of melphalan significantly reduced tumor hypoxia by >99% (*P* = 0.01), with an effect that persisted three weeks after treatment. Scheduled dosing of subconjunctival melphalan (10 mcg given 2x/week for 3 weeks) also decreased tumor hypoxia by 27.9%, although not statistically significant (*P* = 0.36).

**Figure 4:**
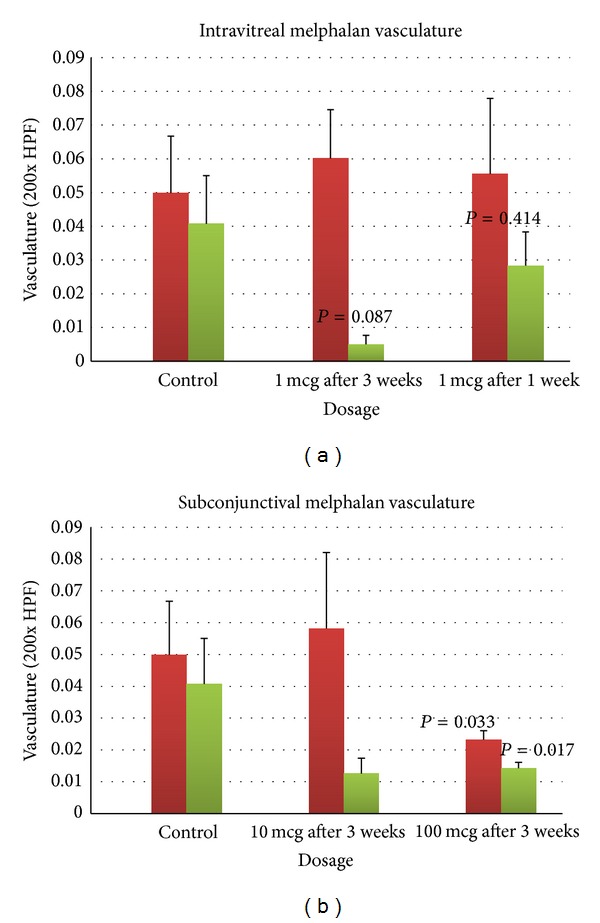
Immature neovessels were reduced following 1 *μ*g of intravitreal injection and following 100 *μ*g of subconjunctival injection, which achieved statistical significance after 3 weeks. Green: neovessels, Red: total vasculature.

**Figure 5:**
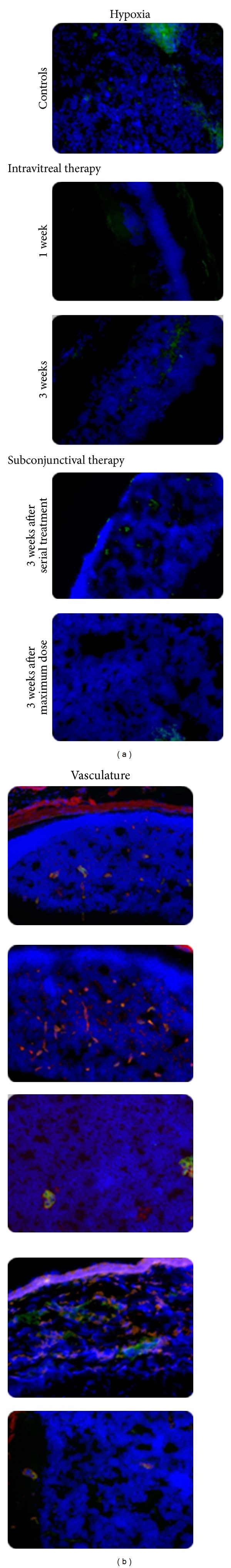
Hypoxia and vasculature reductions after treatment with 1 *μ*g intravitreal injections after 1 week and 3 weeks and following subconjunctival serial injections with 10 *μ*g 2x wk for 3 weeks or a 1-time administration of 100 mcg. Hypoxia showed a statistically significant reduction after treatment with 1 *μ*g intravitreal melphalan at 1 week (*P* = 0.05) and at 3 weeks following a 100 *μ*g maximum dose subconjunctival treatment (*P* = 0.011).* Blue*: DAPI stain for all the cell nuclei;* green*: pimonidazole stain for hypoxic regions. Red (extrAvidin-cy3) stains mature vasculature and green (anti-endoglin) stains neovessels. Pictures were obtained at magnification ×200 high power field.

Regarding the effect of melphalan on tumor vasculature, a decrease in immature neovessels was observed following high-dose subconjunctival melphalan (100 mcg). Using this maximum dosage of subconjunctival melphalan, there was a significant reduction in total and immature vessels compared to controls. After 100 *μ*g of subconjunctival melphalan, total vasculature was significantly reduced by 53.9% (*P* = 0.033), and concentration of neovessels reduced by 65.0% (*P* = 0.017) (Figure 4). Following intravitreal melphalan injections, we did not notice a decline in total vasculature in the treatment arm compared to controls. Immature neovessels declined by 87.7% (*P* = 0.086) demonstrating borderline statistical significance at 3 weeks following 1 *μ*g intravitreal injection (see Figure 5).

## 4. Discussion

In this study, we used a transgenic murine model of retinoblastoma to demonstrate the effects of local treatment with melphalan, both intravitreal and subconjunctival. Melphalan was shown to significantly inhibit tumor growth, decrease tumor vasculature, and reduce hypoxia in vivo when delivered locally. Intravitreal melphalan when given as a single injection reduced tumor burden by 85% at 1 week after injection, with this effect persisting with an 83% reduction 3 weeks after injection. Since the first presentation of this data, small clinical case series have been reported. Our study results mirror early reports regarding intravitreal melphalan in children with advanced retinoblastoma, showing a profound effect on vitreous seeding and globe-conservation [8, 15]. However, reports with intravitreal melphalan in children with retinoblastoma and vitreous seeding utilize 8–30 mcg weekly, with one series showing severe toxicities of cataract, hypotony, vitreous hemorrhage, and subretinal hemorrhage with 50 mcg doses [15]. Munier et al. reported globe-salvage of 87% in 23 patients with advanced retinoblastoma and vitreous seeding treated with 20–30 mcg intravitreal injections of melphalan [14]. Ghassemi and Shields treated 12 children with retinoblastoma and vitreous seeding with doses of 8–50 mcg of melphalan. In patients treated with lower doses (8 and 10 mcg), tumor control was only 43% at long-term (more than 6 months) follow-up. Tumor control was 100% with high-dose (50 mcg) melphalan, but complications and severe toxicities were seen [15]. Regarding the effects on tumor vasculature and hypoxia, intravitreal melphalan was shown to significantly reduce the density of new vessels despite no significant effect on total vasculature. Hypoxia was shown to transiently decrease 1 week after injection with this effect diminishing by 3 weeks. No significant toxicities were seen on histopathologic examination following intravitreal melphalan. However, intravitreal injection in an eye harboring active retinoblastoma sparks considerable controversy amidst concerns of extraocular extension and metastasis. Modified intravitreal injection techniques have been pioneered, combining anterior chamber paracentesis prior to injection to prevent vitreous reflux and injections performed under the operating microscope away from active tumor or vitreous seeds as well as triple freeze-thaw cryotherapy at the injection site to decrease the risk of extraocular spread. Initial reports with these techniques have shown very low risk of metastatic or extraocular spread [18]. However, these risks should be considered and discussed with parents prior to intravitreal treatment. In addition, further studies with additional patients and longer follow-up are needed to determine efficacy and safety.

We have also shown that subconjunctival melphalan is effective in reducing tumor burden in murine retinoblastoma models. There was a significant decrease in tumor burden following serial subconjunctival injections of melphalan, showing an 86% reduction. Additionally, a single subconjunctival injection of 100 mcg of melphalan in advanced tumors resulted in a 91% reduction in the tumor size. Serial subconjunctival melphalan injections did not significantly alter tumor hypoxia, but a single dose of 100 mcg of melphalan did show a significant reduction in tumor hypoxia. These findings suggest that, at higher doses, melphalan may target hypoxic cells via a different mechanism. Additionally, whereas serial, low-dose subconjunctival melphalan injections decreased immature vessels, a single, high dose of melphalan resulted in a reduction of both total and immature vessels. Again, these findings suggest a possible difference in mechanism for high-versus low-dose melphalan treatments. Locally delivered chemotherapy has been used for small retinoblastoma tumors when combined with focal laser therapy as well as an adjuvant to systemic chemotherapy combined with focal laser treatment. Sub-Tenons carboplatin has shown efficacy as a combination therapy but not as monotherapy. Current trials are being conducted by the Children's Oncology Group investigating local chemotherapy. The current study shows the potential of locally delivered melphalan for retinoblastoma and warrants further study.

The advantages of administering chemotherapeutic agents locally, specifically subconjunctival (sub-Tenons') or intravitreal injections, include the ability to administer high concentrations of medication that would otherwise cause considerable toxicity if administered systemically [6, 7]. The usage of intra-arterial melphalan is a clinically practiced therapy for treatment of retinoblastoma [11, 12, 17, 18] but it has been shown to cause multiple adverse effects locally as mentioned earlier [12, 19–23, 36].

Due to the side effects of intra-arterial melphalan delivery, studying alternative routes of local chemotherapy in the form of intravitreal or subconjunctival/sub-Tenon's injections is warranted. One advantage of subconjunctival delivery over intravitreal injection is the decreased risk of tumor dissemination or extraocular spread, although local sub-Tenon's injections do involve a low risk of inadvertent globe perforation [6]. Previous studies using these routes of drug delivery have shown benefits in murine/animal models, where subconjunctival carboplatin injections had a dose-dependent effect on tumor control [6, 7]. These modes of treatment have also been shown to be efficacious in small cases series, but without validation via larger studies. Furthermore, the utilization of animal models before clinical applications remains critical in order to minimize risk, maximize treatment efficacy, and understand drug mechanism of action.

The lack of an ideal animal model poses a challenge in studying new therapies for retinoblastoma. Previous animal models have included nude mouse xenografts [37, 38] and human-adenovirus-type-12-induced rodent tumors [39, 40]. However, both models have substantial anatomic, histopathologic, immunologic, and genetic differences from human retinoblastoma. One advantage of the current study is the use of a transgenic mouse model of hereditary retinoblastoma that has been characterized thoroughly and is remarkably similar to human retinoblastoma in terms of anatomic, genetic, light and electron microscopic, immunohistochemical, and ultrastructural features [25–28]. These similarities, along with the autosomal dominant transmission and high penetrance rate of spontaneous bilateral intraocular tumors, make this an excellent animal model for testing potential treatment modalities [41]. One limitation of the current model is the lack of vitreous tumor seeding. Vascular targeting agents, such as anecortave acetate, have proven efficacious in the *LH*⁡_BETA_T_AG_ mouse model for retinoblastoma, demonstrating a decrease in the vascularity of tumors and enhancing tumor control when combined with chemotherapy or other agents [42]. Glycolytic inhibitors, such as 2-deoxy-D-glucose, have also been investigated in the *LH*⁡_BETA_T_AG_ model and shown to target hypoxic regions of tumors [43]. These novel treatments warrant continued investigation as adjuvant treatments when combined with locally or systemically delivered chemotherapy.

Future studies are needed to explore the impact of locally delivered chemotherapy, including combinations of melphalan, topotecan, and carboplatin [11, 12]. Additionally, ideal dosages must be determined to deliver maximal efficacy while minimizing toxicity. Study limitations include a small sample size and the use of transgenic animal model. However, this is the first study to our knowledge exploring intravitreal and subconjunctival melphalan.

Locally delivered chemotherapy has been shown to be efficacious in treating children with retinoblastoma. Chemotherapy may be delivered via sub-Tenon injection, intravitreal injection, or intra-arterial delivery. The current study provides further support for the efficacy of subconjunctival and intravitreal chemotherapy utilizing melphalan in the treatment of retinoblastoma.
